# Supplementary material for: Role and mechanism of NCAPD3 in promoting malignant behaviors in gastric cancer
Source: Front Pharmacol. 2024 Apr 22;15:1341039. doi: 10.3389/fphar.2024.1341039 (PMC11070777; doi:10.3389/fphar.2024.1341039)
Supplement: Supplementary file 11 [file DataSheet2.ZIP › GSEA/immunologic/my_analysis.Gsea.1599462003817/GSE18791_CTRL_VS_NEWCASTLE_VIRUS_DC_10H_UP.html]

Details for gene set GSE18791\_CTRL\_VS\_NEWCASTLE\_VIRUS\_DC\_10H\_UP[GSEA]

|  || Dataset | filtered\_dataset.sample\_info.cls#WT\_versus\_NCAPD3\_MUT |
| Phenotype | sample\_info.cls#WT\_versus\_NCAPD3\_MUT |
| Upregulated in class | WT |
| GeneSet | GSE18791\_CTRL\_VS\_NEWCASTLE\_VIRUS\_DC\_10H\_UP |
| Enrichment Score (ES) | 0.38850802 |
| Normalized Enrichment Score (NES) | 1.9161775 |
| Nominal p-value | 0.004950495 |
| FDR q-value | 0.58688855 |
| FWER p-Value | 1.0 |
Table: GSEA Results Summary

  

Fig 1: Enrichment plot: GSE18791\_CTRL\_VS\_NEWCASTLE\_VIRUS\_DC\_10H\_UP      
 Profile of the Running ES Score & Positions of GeneSet Members on the Rank Ordered List

  

| SYMBOL | TITLE | RANK IN GENE LIST | RANK METRIC SCORE | RUNNING ES | CORE ENRICHMENT || 1 | 64172 | OSGEPL1 | 6 | 1.152 | 0.0572 | Yes |
| 2 | 222236 | NAPEPLD | 36 | 0.977 | 0.0883 | Yes |
| 3 | 57542 | KLHL42 | 59 | 0.876 | 0.1191 | Yes |
| 4 | 285989 | ZNF789 | 66 | 0.857 | 0.1606 | Yes |
| 5 | 55794 | DDX28 | 68 | 0.856 | 0.2056 | Yes |
| 6 | 7733 | ZNF180 | 154 | 0.728 | 0.1828 | Yes |
| 7 | 11162 | NUDT6 | 162 | 0.716 | 0.2160 | Yes |
| 8 | 57325 | CSRP2BP | 176 | 0.698 | 0.2439 | Yes |
| 9 | 339559 | ZFP69 | 188 | 0.686 | 0.2725 | Yes |
| 10 | 5189 | PEX1 | 200 | 0.676 | 0.3007 | Yes |
| 11 | 55172 | DNAAF2 | 224 | 0.657 | 0.3191 | Yes |
| 12 | 84078 | KBTBD7 | 295 | 0.594 | 0.3000 | Yes |
| 13 | 84640 | USP38 | 303 | 0.589 | 0.3264 | Yes |
| 14 | 79896 | THNSL1 | 407 | 0.534 | 0.2802 | Yes |
| 15 | 54931 | TRMT10C | 446 | 0.507 | 0.2797 | Yes |
| 16 | 54482 | TRMT13 | 449 | 0.506 | 0.3053 | Yes |
| 17 | 5295 | PIK3R1 | 467 | 0.493 | 0.3193 | Yes |
| 18 | 10940 | POP1 | 497 | 0.474 | 0.3236 | Yes |
| 19 | 51112 | TRAPPC12 | 504 | 0.471 | 0.3444 | Yes |
| 20 | 29090 | TIMM21 | 537 | 0.453 | 0.3454 | Yes |
| 21 | 9816 | URB2 | 581 | 0.434 | 0.3374 | Yes |
| 22 | 79697 | C14orf169 | 616 | 0.415 | 0.3350 | Yes |
| 23 | 5147 | PDE6D | 624 | 0.410 | 0.3518 | Yes |
| 24 | 55735 | DNAJC11 | 625 | 0.409 | 0.3736 | Yes |
| 25 | 2021 | ENDOG | 648 | 0.400 | 0.3790 | Yes |
| 26 | 56252 | YLPM1 | 665 | 0.394 | 0.3885 | Yes |
| 27 | 6182 | MRPL12 | 729 | 0.359 | 0.3620 | No |
| 28 | 128061 | C1orf131 | 738 | 0.356 | 0.3752 | No |
| 29 | 51118 | UTP11L | 773 | 0.336 | 0.3685 | No |
| 30 | 29960 | FTSJ2 | 805 | 0.319 | 0.3631 | No |
| 31 | 861 | RUNX1 | 1293 | -0.687 | 0.0466 | No |
| 32 | 64854 | USP46 | 1300 | -0.702 | 0.0798 | No |
Table: GSEA details [plain text format]

  

Fig 2: GSE18791\_CTRL\_VS\_NEWCASTLE\_VIRUS\_DC\_10H\_UP      
 Blue-Pink O' Gram in the Space of the Analyzed GeneSet

  

Fig 3: GSE18791\_CTRL\_VS\_NEWCASTLE\_VIRUS\_DC\_10H\_UP: Random ES distribution      
 Gene set null distribution of ES for **GSE18791\_CTRL\_VS\_NEWCASTLE\_VIRUS\_DC\_10H\_UP**
